# Control of the nanosized defect network in superconducting thin films by target grain size

**DOI:** 10.1038/s41598-021-85304-4

**Published:** 2021-03-16

**Authors:** Moe Moe Aye, Elmeri Rivasto, Mukarram Zaman Khan, Hannes Rijckaert, Esko Salojärvi, Christopher Haalisto, Ermei Mäkilä, Heikki Palonen, Hannu Huhtinen, Isabel Van Driessche, Petriina Paturi

**Affiliations:** 1 Wihuri Physical Laboratory, Department of Physics and Astronomy, University of Turku, 20014 Turku, Finland; 2 University of Turku Graduate School (UTUGS), University of Turku, 20014 Turku, Finland; 3 SCRiPTS, Department of Chemistry, Ghent University, Krijgslaan 281 S3, 9000 Ghent, Belgium; 4 Inorganic Materials Chemistry, Department of Chemistry, University of Turku, 20014 Turku, Finland; 5 Materials Research Laboratory, Department of Physics and Astronomy, University of Turku, 20014 Turku, Finland; 6 Laboratory of Industrial Physics, Department of Physics and Astronomy, University of Turku, 20014 Turku, Finland

**Keywords:** Materials science, Physics

## Abstract

A nanograined YBCO target, where a great number of grain boundaries, pores etc. exist, is shown to hold an alternative approach to future pulsed laser deposition based high-temperature superconductor thin film and coated conductor technologies. Although the nanograined material is introduced earlier, in this work, we comprehensively demonstrate the modified ablation process, together with unconventional nucleation and growth mechanisms that produces dramatically enhanced flux pinning properties. The results can be generalized to other complex magnetic oxides, where an increased number of defects are needed for modifying their magnetic and electrical properties, thus improving their usability in the future technological challenges.

## Introduction

Due to its simplicity and excellent performance on formation of the high density of natural defects, pulsed laser deposition (PLD) is one of the most versatile deposition techniques to get the most effective pinning of flux in high-temperature superconductor (HTS) YBa$_2$Cu$_3$O$_{6+x}$ (YBCO) films^1–3^. However, PLD is quite sensitive to parameters such as target quality, target-to-substrate distance, laser fluence, background pressure and substrate temperature, which can influence the formation of numerous nanoscale defect structures within the superconductor, under the non-equilibrium conditions during the film growth^4–7^. The most critical PLD parameters and their role in defect formation and thus flux pinning have been widely discussed earlier^5,7–15^, but the effect of target quality such as target density and especially its grain size is still not yet completely understood. Especially, since it is traditionally assumed that the laser pulse generally breaks down the target surface at the atomic level and therefore the grain size of the target should not have any effect on the final composition of the deposited film^16,17^.

In previous studies^2,15,18^, the results that show how the target density affects the superconducting properties are evidently inconsistent, but the target density clearly has impact on the surface morphology. On the other hand, it has been found that nanocrystalline PLD target exposed a significant differentiation of the growth kinetics of the laser ablated particles when compared with microcrystalline target, causing smaller growth islands^5^. Moreover, higher defect density and smaller twin domain size have been observed in the films made from nanocrystalline material which leads to a faster relaxation of the lattice with the thickness of the films^5,7,12,14^. As a result, different defect microstructures in YBCO matrix were considered to be the main reason behind the better flux pinning. Therefore, the laser deposited films made from nanocrystalline target can produce effective pinning sites to enhance $J_{\mathrm {c}}$ due to the dense natural defects network^9,11,13^. In order to achieve an intimate knowledge about the influence of PLD target grain size with respect to their density on critical characteristics of YBCO film, the more detailed scrutinies on these characteristics needs to be made.

In this study, pristine YBCO films without artificially produced nanoinclusions are prepared by PLD using microcrystalline and nanocrystalline targets with different target densities. The focus of attention in the main paper is in analyzing the relationship between the target grain size and the film structural characteristics, whereas the superconducting performance of the YBCO films is mainly discussed in the supporting information (SI).

## Results and discussion

### Chemical and morphological properties of the targets

The collection of the structural properties of the $\mu$1-, $\mu$2- and n-YBCO targets are given in Table 1. The measurements of the geometrical densities $\rho _{\mathrm {geom}}$ showed that two clearly different densities, as can be also expected based on the different heat treatments and sintering temperatures, can be obtained for two $\mu$-YBCO targets such as 4.8 and 5.4 g/cm$^3$ for $\mu$1- and $\mu$2-YBCO, respectively. The $\rho _{\mathrm {geom}}$ for n-YBCO falls in between them, being 5.2 g/cm$^3$. On the other hand, the absolute densities $\rho _{\mathrm {abs}}$ are down the line substantially bigger, being 6.17, 6.21 and 5.81 g/cm$^3$ for the $\mu$1-, $\mu$2- and n-YBCO targets, respectively. In the case of equal chemical composition of the material (as shown in SI), a considerably smaller absolute density indicates higher disorder level such as cracks, tortuous passageways or pore spaces inside the sample. The conclusion with the smaller $\rho _{\mathrm {abs}}$ in n-YBCO than in both $\mu$-YBCO targets fits nicely to the structural differences, where the smaller grain size leads to a greater number of grain boundaries in n-YBCO.

**Table 1:** Structural properties of the targets: the geometrical and absolute densities of the targets, lattice parameters, FWHM of the 2$\theta$(005) peaks ($\Delta \theta$) and the target crystallite and grain sizes.

Target	$\rho _{\mathrm {geom}}$ (g/cm$^3$)	$\rho _{\mathrm {abs}}$ (g/cm$^3$)	*a* (Å)	*b* (Å)	*c* (Å)	$\Delta \theta$ ($^\circ$)	Cryst. size (nm)	Grain size ($\upmu$m)
$\mu$1-YBCO	4.8	6.17	3.814	3.883	11.670	0.10	110	5–20
$\mu$2-YBCO	5.4	6.21	3.813	3.884	11.671	0.18	49	5–20
n-YBCO	5.2	5.81	3.859	3.878	11.671	0.23	37	0.1–0.5

**Figure 1:**
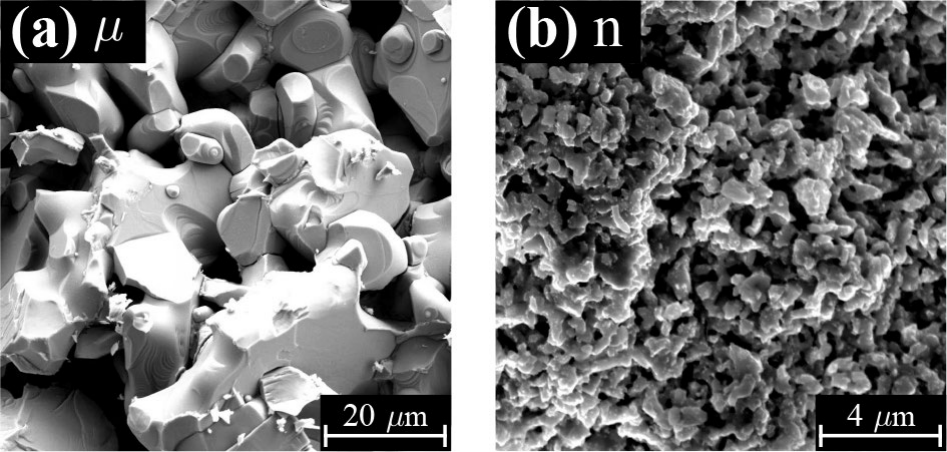
SEM images of the $\mu$- (**a**) and n-YBCO (**b**) target surfaces, which give a clear indication of different grain sizes of the targets (note the different in-plane scales).

The XRD results of the targets are discussed in detail in SI. The Scherrer formula using the FWHM values of the (005) peaks ($\Delta \theta$) is used for calculating an average crystallite size of the targets. This method provides a minimum size of the coherently scattered domains, which again is completely different than the grain size. Since the target annealing procedure and sintering are interpreted as an Ostwald ripening process by nature, the size of the raw material sets the boundaries to the final grain size. Therefore, based on the details in the synthesis and sintering processes, we can estimate that in $\mu$-YBCO target the grain size is in the order of several microns, typically above 5 $\upmu$m, while in n-YBCO the grain size could be readily by two orders of magnitude smaller^19–21^. As can be seen from the SEM images of the targets in Fig. 1, the observed differences in the surface morphology greatly support this result and the grain sizes can be estimated as 100–500 nm for n-YBCO and 5–20 $\upmu$m for $\mu$-YBCO, respectively.

As shown in SI in detail, the difference in surface morphology between micrograined and nanograined targets can also be seen after laser irradiation, which means that the grain size of the target has a remarkable role in the melting and vaporization of the material. This can be qualitatively understood by the model, where relatively smaller energy fluence in n-YBCO target is needed for vaporization, which again leads to a smaller melt depth and a formation of periodic ripples with smaller dimensions. This is also in agreement with our earlier observation, where clearly lower threshold laser energy is needed for evaporating the material from the nanocrystalline than microcrystalline target, thus getting the stoichiometric amount of YBCO elements at the normal deposition distance^5,17,22^.

**Figure 2:**
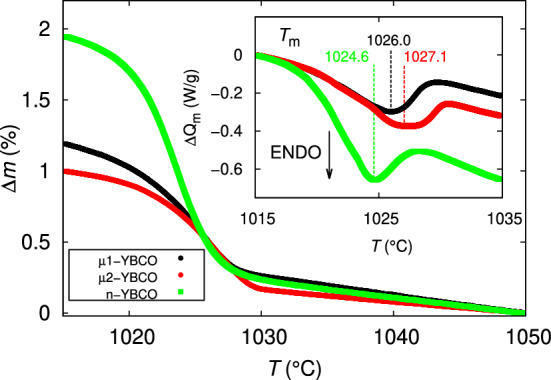
The mass loss step, $\Delta m$, determined during the thermogravimetric analysis (TGA) for $\mu$1-, $\mu$2- and n-YBCO targets. The inset shows a relative change in heat flow per unit mass, $\Delta Q_{\mathrm {m}}$, together with calculated melting points, $T_{\mathrm {m}}$.

Since the surface microstructures of the laser treated targets indicate that the target grain size could have an effect on melting kinetics, we measured the targets by simultaneous thermogravimetry (TG) and differential scanning calorimetry (DSC). The TG mass loss steps together with the heat flow curves are shown in Fig. 2. The heating rate as well as the shape and the size of the sample in each case were kept constant, since they have been observed to have an important effect on mass loss and the shape of the DSC peak^23^. Based on the results, during the endothermic melting process where oxygen evaporates from the YBCO, the mass loss in n-YBCO seems to be almost doubled, $\approx$ 2%, indicating an excess of encapsulated oxygen or a faster release of lattice oxygen through the grain boundaries, pores etc.^24^ of n-YBCO when compared with the values of both $\mu$-YBCO targets. However, based on the XRD measurements and superconductive properties (shown in this paper and SI), we can confirm that the oxygen stoichiometry in the YBCO lattice in all the samples is in practice identical^25,26^. Therefore, the smaller grain size in n-YBCO means a greater number of grain boundaries making n-YBCO thermally less stable, which again could lead to faster release of oxygen. The melting could also assist the more intensive sublimation, which is also supported by the more pronounced endotherm in n-YBCO than in $\mu$-YBCO targets, as indicated by the enthalpies of fusion $\Delta H_{\mathrm {m}}$ of 74.8, 85.7 and 98.5 J/g for $\mu$1-, $\mu$2- and n-YBCO, respectively. For estimating the differences in the melting temperature, $T_{\mathrm {m}}$, between the targets, we have used the peak minimum of the curves, since the discrepancy of onset and endset temperatures have been observed to be significant^23^. As can be seen, $T_{\mathrm {m}}$ is also the smallest in n-YBCO, 1024.6 $^\circ$C, although the difference is not prominent, being 1026.0 $^\circ$C and 1027.1 $^\circ$C in $\mu$1- and $\mu$2-YBCO, respectively. The considerably broader endotherm of n-YBCO, however, suggests the presence of smaller, nanoscale structures, where gradual depression of the melting temperature has been observed with increasing surface-to-volume ratio^27^.

### Crystalline and microstructure of the films

**Figure 3:**
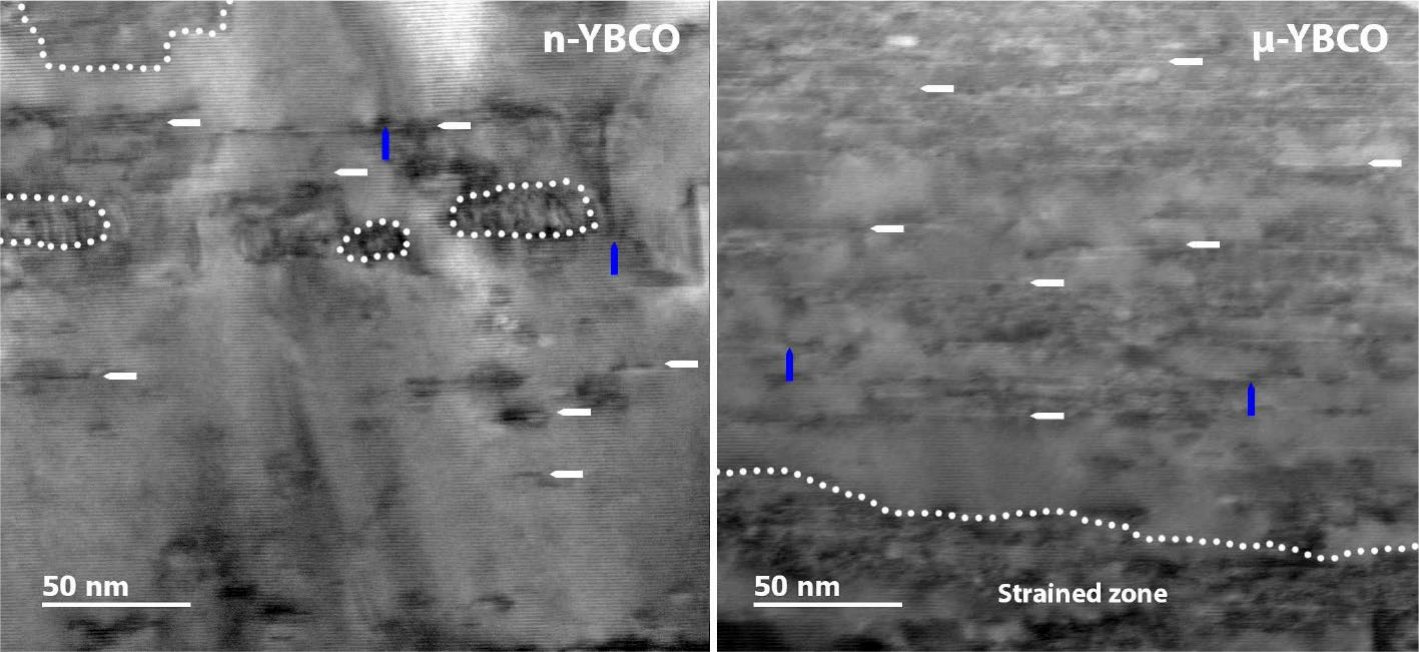
The cross-sectional TEM images of pristine YBCO films deposited from nanocrystalline (n-YBCO) and microcrystalline ($\mu$1-YBCO) targets. White arrows indicate the stacking faults along the *ab*-plane and blue arrows the basal dislocations. The strained zones with Y248 intergrowths are bordered by the dotted line.

As shown in SI, the detailed XRD analysis of the films clearly indicate that, in contrast to $\mu$-YBCO, a great number of defects and dislocations can be expected in n-YBCO, as will be supported by the following TEM analysis. Figure 3 shows the cross-sectional TEM images taken from n-YBCO and $\mu$-YBCO films. In both the films, the most commmon seem to be the planar defects, such as the YBa$_2$Cu$_4$O$_8$ (Y248) intergrowths and stacking faults parallel to the *ab*-plane, where an extra Cu–O chain layer appears between two Ba–O layers^28,29^. In addition, a few regions in the images have a very strong strain contrast with a high concentration of dislocations mostly lying on the basal plane. These could be rendered by stress-related effect at the contact of grain boundaries and the situation can be analogous to the nucleation of intergrowths at grain boundaries^30^. The strained zones, bordered by the dotted line, consist of Y248 intergrowths and these regions can be considered highly localized strained areas with depressed superconducting order parameter, thus being as effective pinning centers^29–33^. In n-YBCO, these regions of Y248 intergrowths are randomly distributed, while in $\mu$-YBCO, Y248 is only observed next to the substrate/film interface. The more and widely distributed strain regions in the n-YBCO can be due to greater number of nucleation sites and smaller growth island size. Hence, n-YBCO shows a greater number of Y248 intergrowths in the range of 15 to 200 nm, while in $\mu$-YBCO, a smaller number of regions in the range of 15–50 nm can be observed. It should be noticed that an isolated Y248 intergrowth with a finite lateral extension is structurally analogous to a Frank loop dislocation, which is an extrinsic stacking fault surrounded by a partial dislocation as observed in the reference^34^. Based on the (*h*00) bright-field TEM analysis (shown in SI), we can conclude that the presence of a great number of Y248 intergrowths together with greater number of twin boundaries in n-YBCO than in $\mu$-YBCO results in a loss of twin boundary coherence along the *c*-axis, which also leads to a greater number of twin intersections and twin tips, typically associated with the formation of substantial strain that, on the other hand, could have a significant role in anisotropic flux pinning^35,36^.

### Angular dependent flux pinning

**Figure 4:**
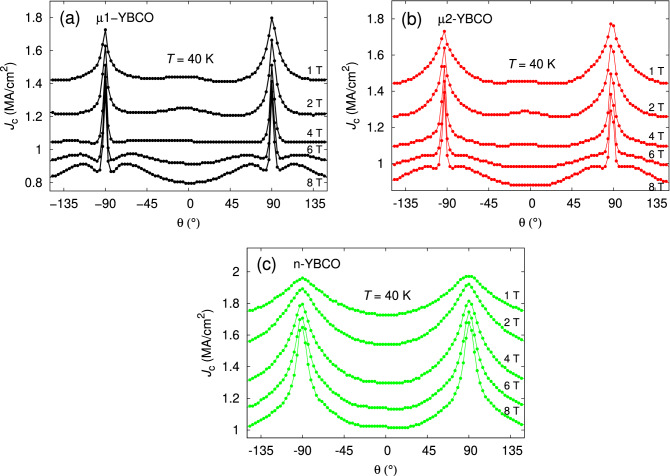
The angular dependencies of $J_{\mathrm {c}}$ measured at 40 K and in wide magnetic field range up to 8 T for undoped $\mu$1-, $\mu$2- and n-YBCO films. The angles $\theta = -90^\circ$ and $90^\circ$ correspond to the *ab*-plane and $\theta = 0^\circ$ the YBCO *c*-axis.

In order to interpret the vortex pinning anisotropy, related to the dimensionality and nature of the defects in $\mu$1-, $\mu$2- and n-YBCO films, the analysis of angular dependent $J_{\mathrm {c}}(\theta )$ with respect to the applied magnetic field and temperature has been performed. The resistively measured critical temperatures were $\approx 91$ K for $\mu$1- and $\mu$2-YBCO films and $\approx 92$ K for n-YBCO film. The $J_{\mathrm {c}}(\theta )$ curves measured at 40 K in wide magnetic field ranges for all three films are shown in Fig. 4a–c. When looking at the shape of the curves, there are no major differences in $J_{\mathrm {c}}(\theta )$ for $\mu$1- and $\mu$2-YBCO, but a notable change can be seen in $J_{\mathrm {c}}(\theta )$ of n-YBCO film. In both microcrystalline films, a relatively sharp *ab*-peak around $\pm \,90^\circ$ can be observed in the entire *B* range up to 8 T. The steep *ab*-peaks can be explained by a great number of *ab*-plane aligned stacking faults and intrinsic CuO planes, which can effectively pin the vortices, especially at high fields^37,38^. However, above 4 T, clear shoulders around the *ab*-peak start to appear. In n-YBCO film, the *ab*-peaks are clearly broader than in both $\mu$-YBCO cases, which could indicate that numerous and widely distributed Y248 intergrowths as well as the strained regions in general not only pin along the *ab*-plane but are also effective in wider angular range in its vicinity^39^. In addition, for both the $\mu$-YBCO films, at $B < 4$ T a small but visible *c*-axis peak can be observed, but not more above 4 T. In n-YBCO film, no remarkable peak along the *c*-axis can be seen. We have earlier explained the appearance of *c*-axis peak in high magnetic field range of pristine YBCO by the presence of great number of twin boundaries and the growth island size related out-of-plane threading dislocations^17^, but the existence of the minor *c*-axis peak in low fields and in $\mu$-YBCO should be understood differently. On this account, the basal dislocations along the *c*-axis observed by TEM can pin the vortices at low fields in both $\mu$-YBCO films, whereas in n-YBCO a great number of stacking faults and randomly distributed Y248 intergrowths lead to shorter pinning distance in the *c*-direction than the standard deviation of the *ab* separation distance of the vortices, thus in agreement with the absence of a *c*-axis peak^17^. When looking at the absolute $J_{\mathrm {c}}$ values, the n-YBCO surpasses both $\mu$1- and $\mu$2-YBCO films in the whole magnetic field and angular ranges. This is a clear proof that the collective pinning structure together with the dense network of twin boundaries and growth island related threading dislocations in n-YBCO film are of unmatched quality, at least at low temperatures.

**Figure 5:**
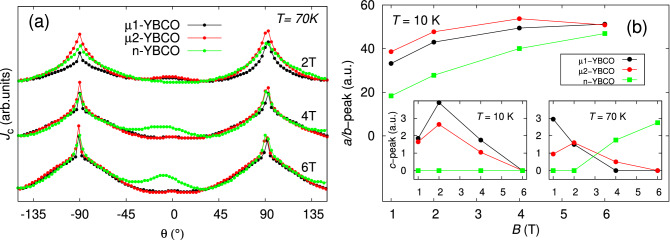
(**a**) The angular dependencies of $J_{\mathrm {c}}$ measured at 70 K and in 2 T, 4 T and 6 T magnetic fields for $\mu$1-, $\mu$2- and n-YBCO films. The angles $\theta = -90^\circ$ and $90^\circ$ correspond to the *ab*-plane and $\theta = 0^\circ$ the YBCO *c*-axis. The curves are shifted in y-direction for detailed shape comparison. (**b**) The relative heights of the *ab*-peaks (the main panel) and *c*-peaks (insets) as the functions of the applied magnetic field in $\mu$1-, $\mu$2- and n-YBCO films.

To get better understanding about the shape and anisotropy differences as well as the temperature evolution of $J_{\mathrm {c}}(\theta )$, we have plotted 70 K data at the fields of 2 T, 4 T and 6 T as shown in Fig. 5a, where the lowest point of each curve was shifted to the same level to make the comparison easier. As can be seen, the shape of the *ab*-peak of both $\mu$-YBCO films approaches that of n-YBCO, being clearly different than the form at low temperatures. In addition at 70 K, the *c*-peak in n-YBCO overtakes the *c*-peak of $\mu$-YBCO, when the external magnetic field $B \ge 4$ T. The rise of the *c*-peak at higher temperatures can be attributed to an increased stability of the vortex lattice. At high temperatures, the vortex lattice is less rigid and thus the increased vortex movement caused by the thermal motion increases the probability of the vortices to collide with the pinning centers. Therefore, the increased pinning probability is responsible for increased $J_{\mathrm {c}}$ along the YBCO *c*-axis, where the growth island related threading dislocations are situated.

Supporting the information of pinning structure even more, we used the $J_{\mathrm {c}}(\theta )$ data to calculate the relative heights of the *ab*- and *c*-peaks as a function of the applied magnetic field. The relative heights of each peak are defined as a difference between the $J_{\mathrm {c}}$ along the YBCO *c*-axis or *a*/*b*-plane and the smallest $J_{\mathrm {c}}$ value within the whole angular range. Although the lowest $J_{\mathrm {c}}$ does not fully correspond to the level that can be calculated from the Blatter scaling^37^, the relative heights are still comparable with each other. Typically, the maxima of these curves give information about the number of dislocation related matching field $B_{\phi }$ in a specific direction, where the number of vortices and effective pinning centers are equal^40^. As can be seen in Fig. 5b, already at 10 K, *ab*-peaks reach their maxima in $\mu$-YBCO films at as low as 4 T, while in n-YBCO the height of the *ab*-peak still increases clearly at 6 T. On the other hand, at 10 K the maxima of *c*-peaks in $\mu$-YBCO films are around 2 T, while in n-YBCO the peaks’ height remains zero. When looking at the situation at a high temperature of 70 K, the maxima in both $\mu$-YBCO films are below 2 T, while in n-YBCO the maximum has recently turned up, being decidedly above 6 T. This is a clear indication of a great number of *c*-axis aligned pinning centers such as dislocations in n-YBCO. Regardless of the target grain size, however, the decreased effective $B_{\phi }$ with increased temperature can be explained by the decreased number of pinned vortices caused by the increased thermal motion. All the results under discussion are in good agreement with the previously discussed and substantially enhanced vortex pinning structure in n-YBCO. The effect of target grain size on the growth of dense nanosized defect network and its importance for flux pinning will be discussed in the following section in detail.

### Target grain size dependent laser interaction

**Figure 6:**
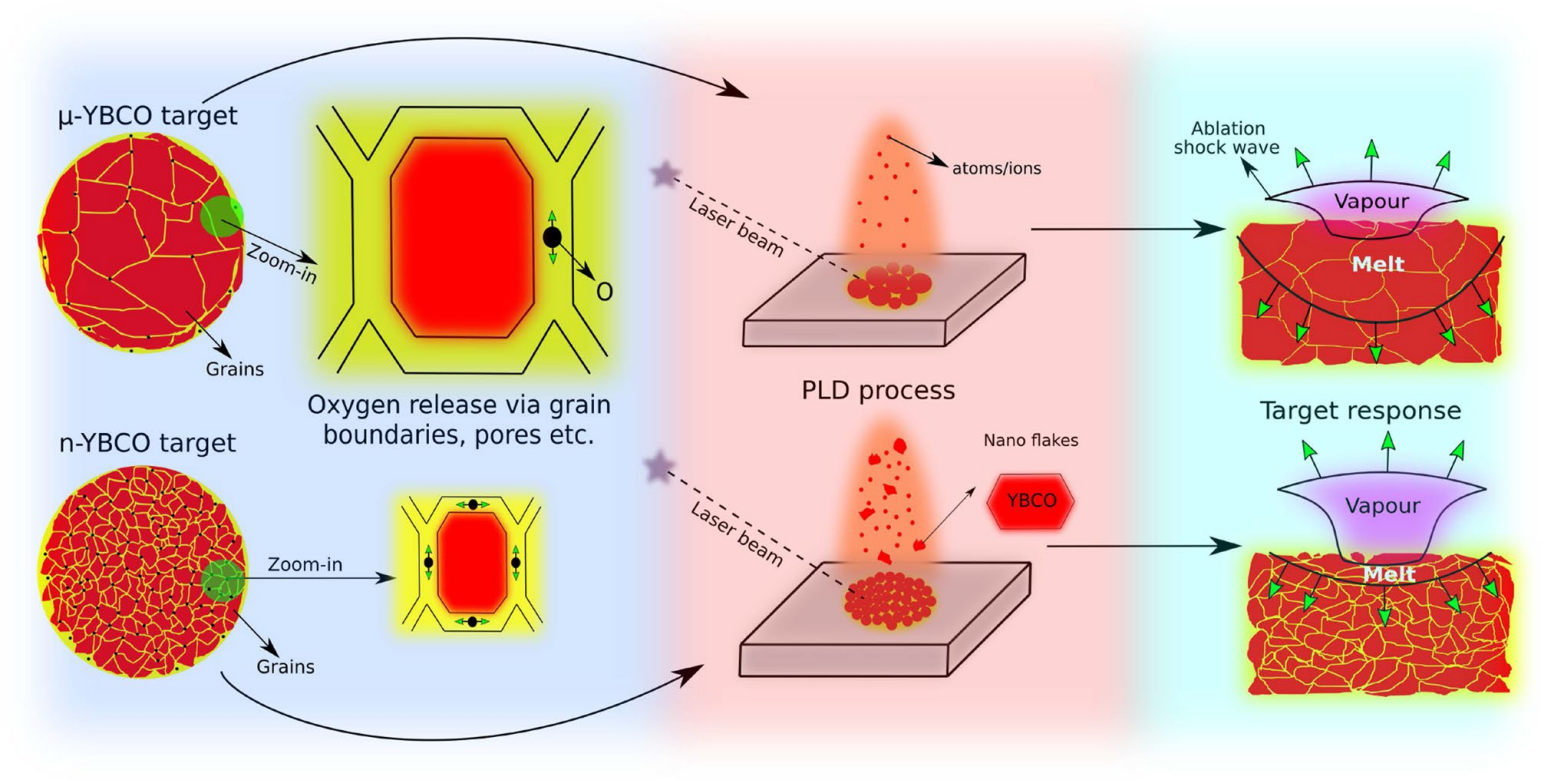
A proposed schematic illustration how nanograined and micrograined YBCO targets lead to different ablation mechanisms. The original target grain size together with differences in the number of grain boundaries and through the grain boundaries released oxygen (on the left) affect the modified laser–target interaction. Since the laser energy is concentrated on the surface layer in n-YBCO target, the melting process is faster, leading to more powerful removal of YBCO (on the right). These mechanisms result in a smooth plume of individual atoms and ions in $\mu$-YBCO, while in n-YBCO a great number of larger fragments of YBCO nanoparticles occur (in the middle).

A deduction chain, how the nanograined target can be proposed as a stepping stone for the future high capability HTS thin films is schematically illustrated in Fig. 6. The model of ablation can be first divided into two phases: the cumulative laser irradiation together with a thermal response of the micrograined and nanograined targets cause a completely different vaporization mechanism and, on the other hand, the nucleation and growth of the basic elements on the surface of the heated substrate.

A small general grain size and a dense network of grain boundaries with for instance pores in n-YBCO target, where a substantial amount of released oxygen can appear, lead to a different diffusion distances and optical absorption depths, when the thermal pulse penetrates the solid. Since the optical absorption coefficient is typically higher in nanograined material^41^, the laser fluence can be assumed more surface sensitive, which again increases the energy density in the surface region, where the thermal diffusivity controls the heating and ablation characteristics^42^. As the energy density is increased, the sub-surface temperature may exceed the surface temperature^43^, leading to rapid surface evaporation. This again could cause an explosive removal of YBCO nanoparticles with high kinetic energy, together with strongly released oxygen from the nanograined n-YBCO. Since the original YBCO particles can be as small as only 1–3 unit cells thick and a few tens of nanometers in diameter^5,19^, they could attain velocity high enough to travel through the background gas. In the case of $\mu$-YBCO, however, the optical absorption length is much higher than the thermal diffusion length and thus the ablation is more related to volume ablation^44^. The volume type ablation is typically understood by the internal superheating, which during the propagation of the ablation front forms a sub-surface gaseous phase^44^. Since the degree of superheating is shown to increase rapidly with decreasing absorption coefficient^44^, we can assume that this type of ablation process is more suitable for removing atoms, ions and molecules as expected in the case of $\mu$-YBCO. The described mechanisms are in line with our earlier observations, where more than five times higher threshold energy is needed for removing the material from $\mu$-YBCO than from n-YBCO target^11^ and also supported by the result, where a greater absorption coefficient decreases the energy density needed for ablation^16^.

The above mentioned laser–target interaction leads to a different composition of the laser plume, when it propagates through the background gas in the cases of micro- and nanograined targets, as presented in Fig. 6. In the microcrystalline $\mu$-YBCO, a smooth plume of mainly individual atoms and ions expands as a shock wave propagation when its pressure exceeds the pressure of the background gas^45^. On the other hand in n-YBCO, the localized laser pulses with distinctly high temperature certainly produce higher kinetic energy to transport the heavier fragments such as voluminous YBCO nanoparticles to the substrate.

### Nucleation and growth model

To understand how various particle size distributions in $\mu$-YBCO and n-YBCO plumes can produce different island sizes in deposited films, we have calculated the density evolution of the growth islands after a single laser pulse by a molecular dynamics simulation, as explained in detail in SI. The difference in the source information of the simulation between $\mu$-YBCO and n-YBCO is culminated in the average differences in the particle size and mass, where the $\mu$-YBCO mainly consists of atoms and ions, while in n-YBCO also a significant number of particles are in the form of heavier fragments^17^.

**Figure 7:**
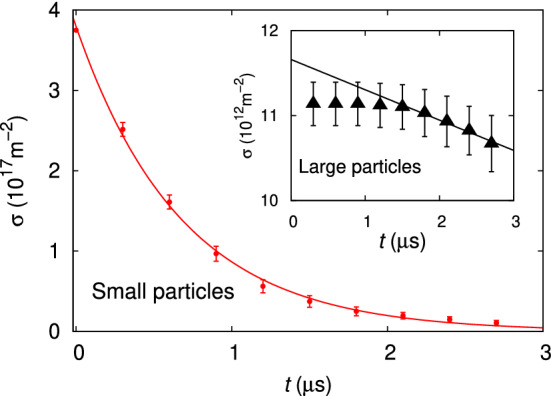
The simulated growth island densities with standard errors as a function of time for small and large particles as in the case of $\mu$-YBCO and n-YBCO, respectively. The solid lines are fits to the exponential (small particles) and linear (large particles) functions.

**Figure 8:**
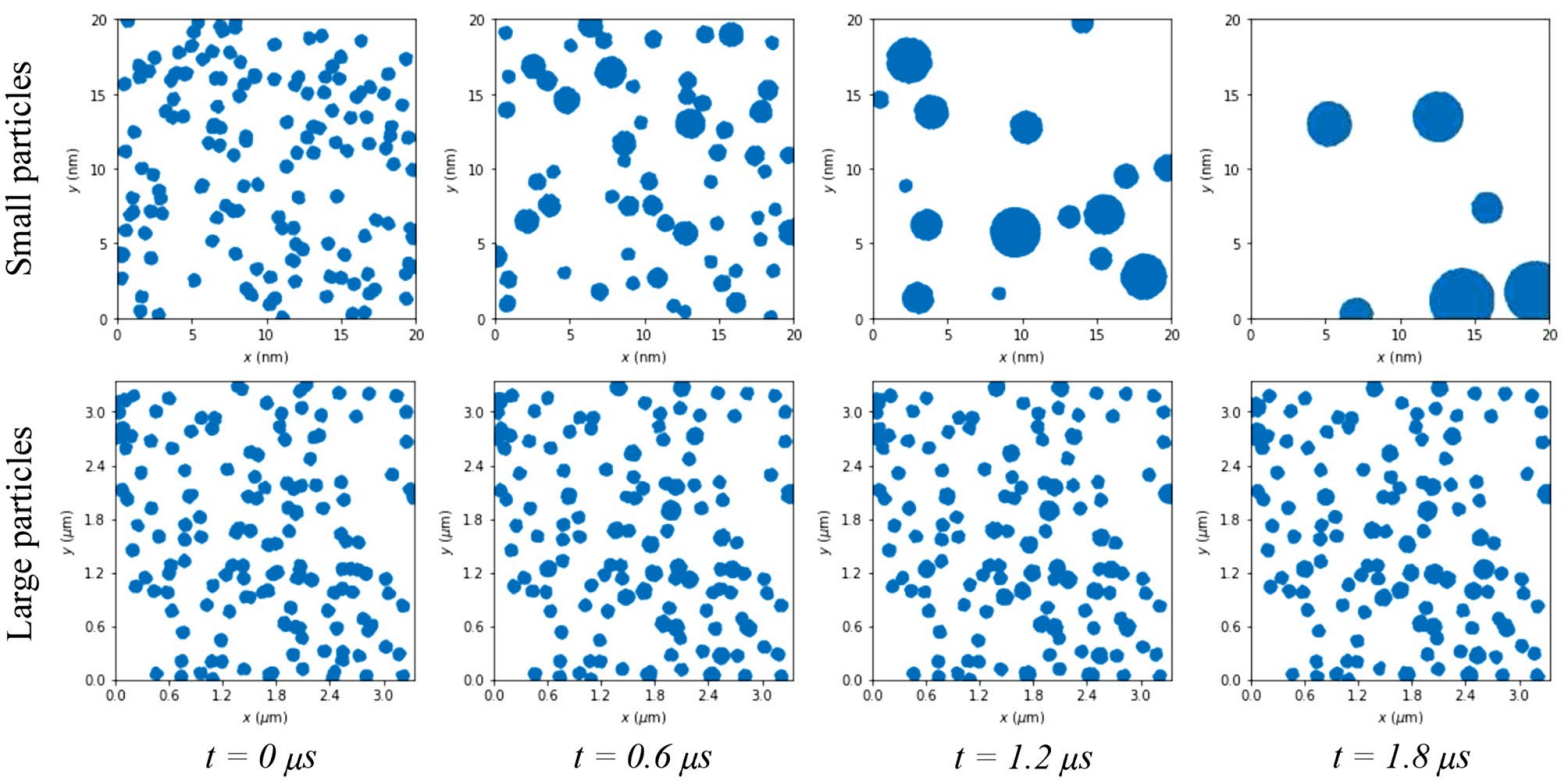
Evolution of the simulated particle densities after different time steps for small and large particles, showing its effect on the island sizes in the cases of $\mu$-YBCO and n-YBCO, respectively.

As can be seen in Fig. 7, in the case of the small particles, as in $\mu$-YBCO, the density of the individual particles decreases exponentially with time, increasing clearly the size of the nucleation centers, while in the case of larger particles (n-YBCO), after a threshold time scale, the density of the growth centers decreases only moderately, being linear in nature. The results are qualitatively explained by the reduced thermal motion of the larger particles due to their superior mass when compared with the smaller ones. The basic idea about the evolution of the particle densities at different time steps, leading finally to the modified growth island size, is illustrated in Fig. 8. The simulations are perfectly in line with our earlier assumptions, where the growth islands in $\mu$-YBCO grow at the end larger than in the case of n-YBCO. This is also in agreement with previous hypothesis, where the diameter of the surface particles of the films as 300 nm in $\mu$-YBCO and 180 nm in n-YBCO correlates with the island size and is therefore related to the density of the threading dislocations, directly linked to the flux pinning^17^.

### The effect of target grain size on critical current density

**Figure 9:**
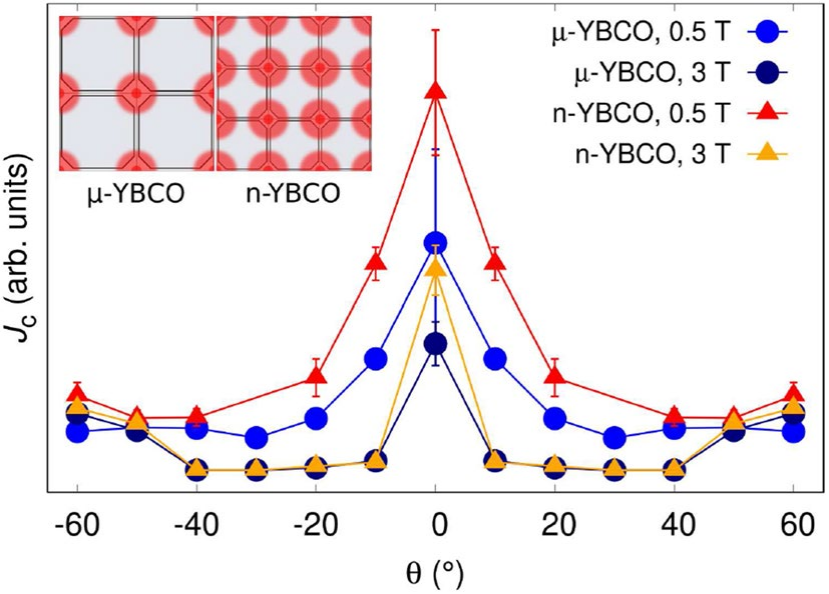
Simulated $J_{\mathrm {c}}(\theta )$ datapoints for $\mu$-YBCO and n-YBCO in two magnetic fields of 0.5 and 3 T. $J_{\mathrm {c}}$ values are given in arbitrary units but the absolute levels of the curves are comparable with each other. The inset shows the schematic networks of growth islands with different diameters in $\mu$-YBCO and n-YBCO films, which produce different densities of threading dislocations along the YBCO *c*-axis. In the simulations, the only difference between the samples is the number of threading dislocations (the diameter of 1 nm), three times more in n-YBCO than in $\mu$-YBCO.

In order to get better understanding how smaller growth island size and thus a greater number of threading dislocations can affect the improved flux pinning mechanism in n-YBCO, we have calculated the effect of dislocation density on the angular dependent critical current density $J_{\mathrm {c}}(\theta )$ using molecular dynamics simulations^17,40,46^. Based on the differences in the growth island sizes in $\mu$-YBCO and n-YBCO, the different number of threading dislocations (three times more in n-YBCO than in $\mu$-YBCO, inset of Fig. 9), were taken into account by simulating them as continuous nanorods with diameter of 1 nm at two different magnetic fields of 0.5 and 3 T. The details of the simulations are given in the SI. As shown in Fig. 9, the effect of dislocation density on the angular dependent $J_{\mathrm {c}}$, especially in the vicinity of *c*-axis, is indisputable. In the low magnetic field range of 0.5 T, the *c*-axis peaks of both $\mu$-YBCO and n-YBCO are relatively sharp with broadened shoulders on the sides, but the peak is obviously more intense in n-YBCO, where more threading dislocations occur. At the high fields of 3 T, the difference in $J_{\mathrm {c}}$ is similar although the *c*-axis peaks are clearly narrower without broadened shoulders. Since the simulations predict that well-aligned dislocations along the YBCO *c*-axis produce relatively narrow *c*-axis peaks, we can qualitatively assume that this is also the case in our thin films, where much sharper *c*-axis peak is visible for n-YBCO film, especially at high temperatures. If thinking about the other possible defects observed by TEM, such as intergrowths, stacking faults, basal dislocations, only the greater number of twin intersections in n-YBCO can additionally be the cause of the similar type of *c*-axis peak formation. Therefore, we can conclude that in addition to improved flux pinning by other defects in n-YBCO film, which increases the general level of $J_{\mathrm {c}}(\theta )$ in wide angular range, the smaller growth island size can play an important role in the flux pinning property, especially along the *c*-axis direction.

### Conclusion

The effect of target density and its crystal grain size on the critical current density $J_{\mathrm {c}}$ of the pulsed laser deposited YBCO thin films is systematically investigated in wide temperature, magnetic field and angular ranges. The absolute value of $J_{\mathrm {c}}$ and its anisotropy are obtained to be similar in the case with different target densities whereas the target grain size is observed to have a great impact on the flux pinning and vortex dynamics, producing a completely different shape of $J_{\mathrm {c}}(\theta )$ curve, especially around the YBCO *a*/*b*- and *c*-axes. The results are explained by the modified deposition process, where the decreased grain size of the target leads to a great number of grain boundaries and thus a modified melting procedure of the target. The explosive removal of heavier nanofragments together with neutral and ionized species in the laser plume leads to a significant number of extra individual nucleation centers on the surface of the substrate. This is expected to change the growth mechanism on the substrate and therefore produce an altered distribution of various types of naturally formed nanosized defects that can act as vortex pinning centers within the YBCO lattice. Based on the angular dependent $J_{\mathrm {c}}$ data and the general understanding about the growth mechanism, we have proposed a model how the different target grain size can result in an increased absolute $J_{\mathrm {c}}$ and modified anisotropy of $J_{\mathrm {c}}$ in the wide angular, magnetic field and temperature ranges. The results obtained here can also be utilized to improve the properties in the wide range of complex magnetic oxides required in the future technologies.

## Methods

### Preparation of micro- and nanograined materials

In order to get microcrystalline ($\mu$1- and $\mu$2-YBCO) targets with different densities, a solid state reaction method was carried out by using commercial grade micron size Y$_2$O$_3$, BaCO$_3$ and CuO raw powders. The stoichiometric quantities of high-purity powders were ground thoroughly and pelletized. Initially, the heat treatment for the powder was done at 950/920 $^\circ$C ($\mu$1/$\mu$2) for a period of 24 h in air. Then, the same process was repeated by performing the grinding-pelletizing procedure and then sintered at the same temperatures for another 24/48 h. The final annealing was carried out in an atmosphere of oxygen at 500 $^\circ$C for 24 h to obtain the micro grain size YBCO target. For nanocrystalline (n-YBCO) target, a citrate-gel method was used. Water solutions of high-purity nitrate salts of Y, Ba, Cu and citric acid were used for the precursor powder preparation. Firstly, the mixture solution was evaporated at 80 $^\circ$C to transform a gel. To get the precursor powder, the gel was heated slowly up to 550 $^\circ$C. Then, the precursor powder was calcinated in flowing air at 830 $^\circ$C for 15 h, then cooled down to room temperature and pressed into a pellet. Finally, the pellet was sintered for 2 h at a relatively low temperature of 900 $^\circ$C, annealed at 500 $^\circ$C for 30 h in an atmosphere of oxygen to get a nano grain sized YBCO target. More details of the target preparation can be found in Ref.^20,47^. The geometrical densities of the targets were determined by a measurement of the amount of mass per unit of volume and the true densities were measured with a He pycnometer (AccuPyc 1330, Micromeritics Instrument Corp.). The YBCO thin films were prepared by PLD using microcrystalline target ($\mu$1- and $\mu$2-YBCO) and nanocrystalline target (n-YBCO) with different target densities. The films were grown on (100) SrTiO$_3$ (STO) substrates using a 308 nm XeCl excimer laser in optimized deposition conditions. The details of the PLD system together with deposition parameters have been given elsewhere^48^.

### Structural characterizations

The surface morphology and elemental composition of the targets was examined by Scanning Electron Microscopy, SEM (FEI Apreo S field-emission SEM with Schottky-type electron gun, Thermo Fisher Scientific) and Energy Dispersive Spectroscopy, EDS (Ultim Max 100 SDD, Oxford Instruments). The EDS elemental maps were taken with 10 kV acceleration voltage and 0.80 nA beam current. The thermal changes of the complexes in different targets were studied with a TA Instruments SDT Q600 simultaneous TGA-DSC apparatus between 800 and 1200 $^\circ$C in flowing synthetic air. The flow rate of the gas was 100 mL/min and all the samples were heated with a rate of 1 K/min. The crystallographic properties of the targets and films were determined by x-ray diffraction (XRD) measurements with a Philips Empyrean system. The phase purity and the lattice parameters were determined from ($\theta ,2\theta$) scans in the (00*l*) direction and from detailed 2D ($\phi ,2\theta$) texture scans of the YBCO (212)/(122) peaks, respectively. The out-of-plane crystallographic texture was determined by XRD rocking curves (RC) of the YBCO (005) peaks ($\omega$ scans). The microstructure of the films as well as the defect formation was investigated with JEOL JEM 2200FS bright-field transmission electron microscopy (BF-TEM) using 200 kV operating voltage. The samples for BF-TEM were prepared by cutting a cross-sectional lamella via the focused Ion beam (FIB) technique in a FEI Nova 600 Nanolab Dual Beam FIB-SEM, using the in situ lift out procedure with an Omniprobe extraction needle^49^.

### Superconducting characterizations

The angular dependent transport properties of all the films were measured by using a horizontal rotator option available in PPMS. The measurements were done at 0.5 T, 1 T, 2 T, 4 T, 6 T and 8 T fields and temperatures of 10 K, 40 K and 70 K with 0$^\circ$ to 360$^\circ$ angular range using 3$^\circ$ of steps. For this purpose, all the films were patterned by wet chemical etching. The etched patterns were 50 $\upmu$m wide current stripes on each film. The contacts on the films were made by aluminium wire using TPT HB05 Wire Bonder.

### MD simulations

Both the nucleation and growth of the YBCO films and the angular dependency of the critical current density $J_{\mathrm {c}}(\theta )$ were modeled by molecular dynamics (MD) simulations. Details of the simulation models are given in Supplementary information (SI).

## Supplementary information

Supplementary informations.
