# Supplementary material for: Control of the nanosized defect network in superconducting thin films by target grain size
Source: Sci Rep. 2021 Mar 16;11:6010. doi: 10.1038/s41598-021-85304-4 (PMC7966807; doi:10.1038/s41598-021-85304-4)
Supplement: Supplementary file 1 — Supplementary informations. [file 41598_2021_85304_MOESM1_ESM.pdf]

Supplementary Information: Control of the nanosized defect network in superconducting thin films by target grain size

Moe Moe Aye[1,2,*], Elmeri Rivasto[1,2], Mukarram Zaman Khan[1,2], Hannes Rijckaert[3], Esko Salojärvi[4], Christopher Haalisto[5], Ermei Mäkilä[6], Heikki Palonen[1], Hannu Huhtinen[1], Isabel Van Driessche[3], and Petriina Paturi[1]

[1]Wihuri Physical Laboratory, Department of Physics and Astronomy, University of Turku, FI-20014 Turku, Finland
[2]University of Turku Graduate School (UTUGS), University of Turku, FI-20014 Turku, Finland
[3]SCRiPTS, Department of Chemistry, Ghent University, Krijgslaan 281 S3, 9000 Ghent, Belgium
[4]Inorganic Materials Chemistry, Department of Chemistry, University of Turku, FI-20014 Turku, Finland
[5]Materials Research Laboratory, Department of Physics and Astronomy, University of Turku, FI-20014 Turku, Finland
[6]Laboratory of Industrial Physics, Department of Physics and Astronomy,University of Turku, FI-20014 Turku, Finland
[*]moe.m.aye@utu.fi

1 Crystallographical and morphological properties of the targets

Based on the XRD (θ, 2θ) scans, all the YBCO targets are nearly phase pure although minor peaks, indicating CuO and $BaCuO_2$ impurities, can be seen (Fig. S1). Similar XRD patterns with split diffraction peaks in both μ-YBCO targets indicate greater crystallite size than in n-YBCO target, where the peaks are significantly broader. The orthorhombicity can also be obtained by looking at the difference between the a and b lattice parameters of the targets (see Table 1 in the main paper).

The surface morphology of the targets after the laser deposition is measured by SEM and the surface images of μ1-, μ2- and n-YBCO targets after laser irradiation are shown in Fig. S2. As can be seen, the surface in both micrograined targets is very similar with irregular surface structure, where the height differences of the surface features are substantial. In addition, the relatively large scale capillary waves of laser cones can be observed. When looking at the surface of the n-YBCO target in Fig. S2(c), the surface seems to be much smoother with clearly smaller and periodic cone ridges. On this account, it seems that although the target density does not affect on the steady-state pattern of the surface, the grain size of the target has a remarkable role in the melting and vaporization of the material. This is not completely in line with an earlier assumption, where the readily observable target properties have not been assumed to affect on the surface cone morphology or the vaporization rate[S1]. As shown in the relative elemental concentration analysis in the table of Fig. S2, the smaller target density in μ1-YBCO produces an almost changeless metal stoichiometry on the target surface after the laser deposition, whereas with the denser microcrystalline target μ2, less Y and more Ba is available than expected for the YBCO with correct nominal stoichiometry. However, since we do not know how this will affect on the crystallization and growth of thin film, we should study the stoichiometry and possible impurity phases in the final thin films. Completely different behaviour can be seen for the n-YBCO target, where the laser thermal response to the target clearly modifies the elemental ratios of Y, Ba and Cu.

2 Crystalline and microstructure of the films

As shown in Fig. S3, all the films show strong (00l) peaks, indicating epitaxially textured and c-axis oriented YBCO phase without clear impurity peaks. In addition, based on the 2θ–ϕ scans of the YBCO (102) peak, there were no a-axis oriented

Table S1. Crystallographical properties of the μ1-, μ2- and n-YBCO films determined by XRD measurements.

Film	a (Å)	b (Å)	c (Å)	$\Delta\theta$ (°)	$\Delta\omega$ (°)	r_c (nm)
μ1-YBCO	3.814	3.878	11.678	0.11	0.27	16.1
μ2-YBCO	3.814	3.878	11.673	0.10	0.27	15.8
n-YBCO	3.804	3.877	11.711	0.22	0.38	11.4

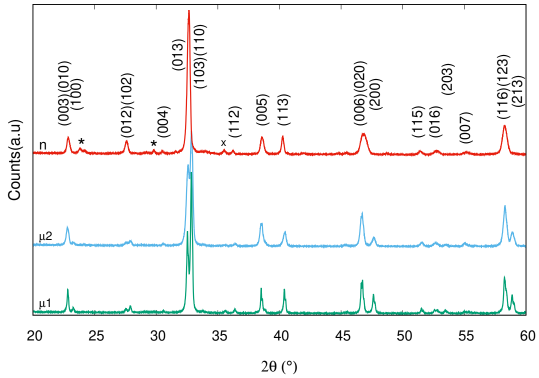

Figure S1. The room temperature x-ray 2θ diffractograms of the μ1-, μ2- and n-YBCO PLD targets.The YBCO peaks were indexed and the star and the cross symbol marks minor peaks associated with the reflection of $BaCuO_2$ and CuO impurity phases, respectively.

Target	Y (at.%)	Ba (at.%)	Cu (at.%)
μ1-YBCO	14.4 / 14.6 (+0.2)	40.1 / 39.8 (–0.3)	45.5 / 45.7 (+0.2)
μ2-YBCO	14.3 / 16.1 (+1.8)	40.1 / 38.5 (–1.6)	45.5 / 45.3 (–0.2)
n-YBCO	16.3 / 14.3 (–2.0)	38.4 / 42.6 (+4.2)	45.2 / 43.1 (–2.1)

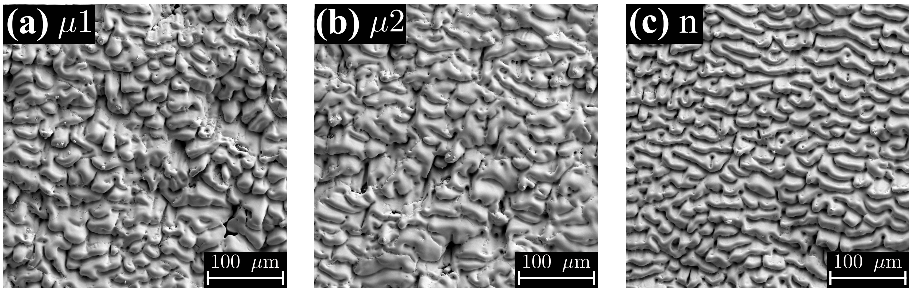

Figure S2. SEM images of the μ1- (a), μ2- (b) and n-YBCO (c) target surfaces after applying the laser pulses in the deposition. Table: the elemental amounts of Y, Ba and Cu in atomic percentages calculated from the relative elemental analysis of YBCO targets taken from the original untreated (left) and evaporated spots (right). The values in the parentheses present the change of elemental concentration on the surface of the target before and after the deposition.

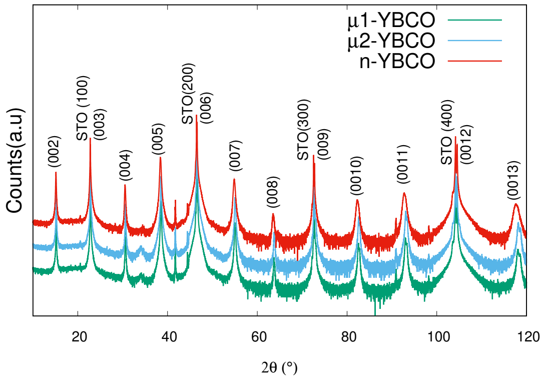

Figure S3. The room temperature x-ray 2θ diffractograms of the μ1-, μ2- and n-YBCO films.

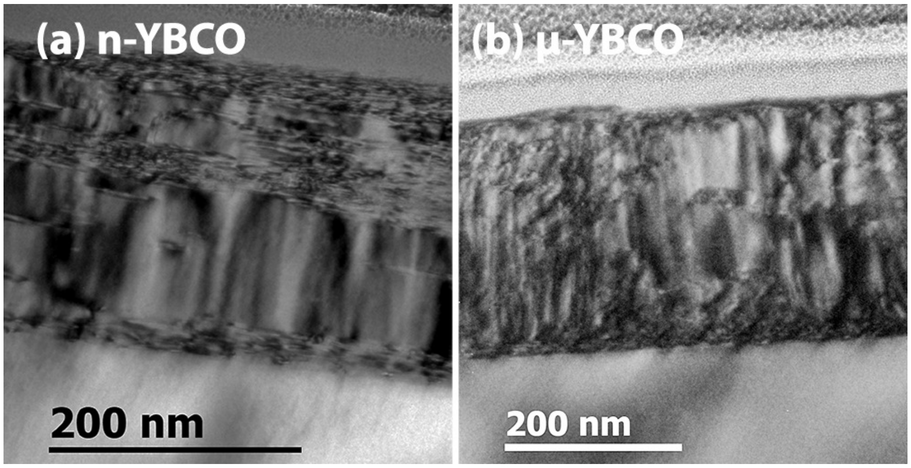

Figure S4. Cross-sectional BF-TEM images indicating a slightly different twin boundary structure in n-YBCO and μ1-YBCO films.

grains in any of the films. In general, the 2θ peaks of n-YBCO are broader and shifted to the slightly lower values when compared with both μ-YBCO films. These observations can be linked to the increase of microstrain and to the variation of the c-axis parameter[S2], being also in line with the lengthened c-axis of n-YBCO (Table S1). The FWHM of the rocking curve of (005) peak $\Delta\omega$ shows that the peak is broader in n-YBCO when compared with the values similar with each other for both μ-YBCO films. Relatively larger $\Delta\omega$ in n-YBCO can be attributed to the deviation of unit cell alignment along the YBCO c-axis that increases the out-of-plane mosaic spread of the film[S3]. On the other hand, when compared with μ-YBCO films, the long range lattice ordering r_c[S4] is clearly decreased in n-YBCO, being perfectly in line with the modified growth, where a great number of disorders and dislocations were formed, as will be discussed in detail in the main paper and SI.

The (h00) bright-field TEM images in Fig. S4(a) and (b) show twin boundaries in both n-YBCO and μ1-YBCO films. The density of the twin boundaries can be estimated to be higher in n-YBCO films, since an average distance between the boundaries, the so-called twin spacing, has been calculated to be 28.5 ± 4.5 nm in n-YBCO and 33.7 ± 6.7 nm in μ1-YBCO, respectively. The results are in good agreement with our earlier observations, where the faster development of twin structure during the growth process and the smaller twin domain size is observed in n-YBCO films[S5,S6].

3 Angular dependent flux pinning

We have compared the absolute J_c values along the YBCO ab-plane and c-axis by plotting the magnetic field dependent J_c values at temperatures of 10 K, 40 K and 70 K (Fig. S5). It can be easily seen that the $J_c(ab)$ at 10 K and 40 K is clearly higher in n-YBCO than in both μ-YBCO films within the whole magnetic field range. In addition, the field dependent decrease of J_c seems also to be smaller in n-YBCO. In spite of the visible c-axis peak at low fields in both μ-YBCO films, n-YBCO still has greater $J_c(c)$ in the entire field range. When looking at the temperature evolution of J_c, we can also obtain that although the general difference of absolute J_c between n-YBCO and μ-YBCO at 70 K approaches each other, the difference between $J_c(ab)$ and $J_c(c)$ in n-YBCO shrinks, being well in line with the pronounced c-axis peak observed in n-YBCO at high fields and temperatures.

4 Principles for simulating the nucleation and growth

The calculations of growth island densities were based on a molecular dynamics simulation, where the circle-shaped particles were allowed to move on a 2D surface according to the Verlet algorithm. The effect of temperature was implemented inside the algorithm via a Langevin thermostat, which produces a temperature dependent random force on the particles making them to randomly walk on the grid. The general iteration algorithm of a single particle located at position $\mathbf{r}_i$ with velocity $\mathbf{v}_i$ at iteration

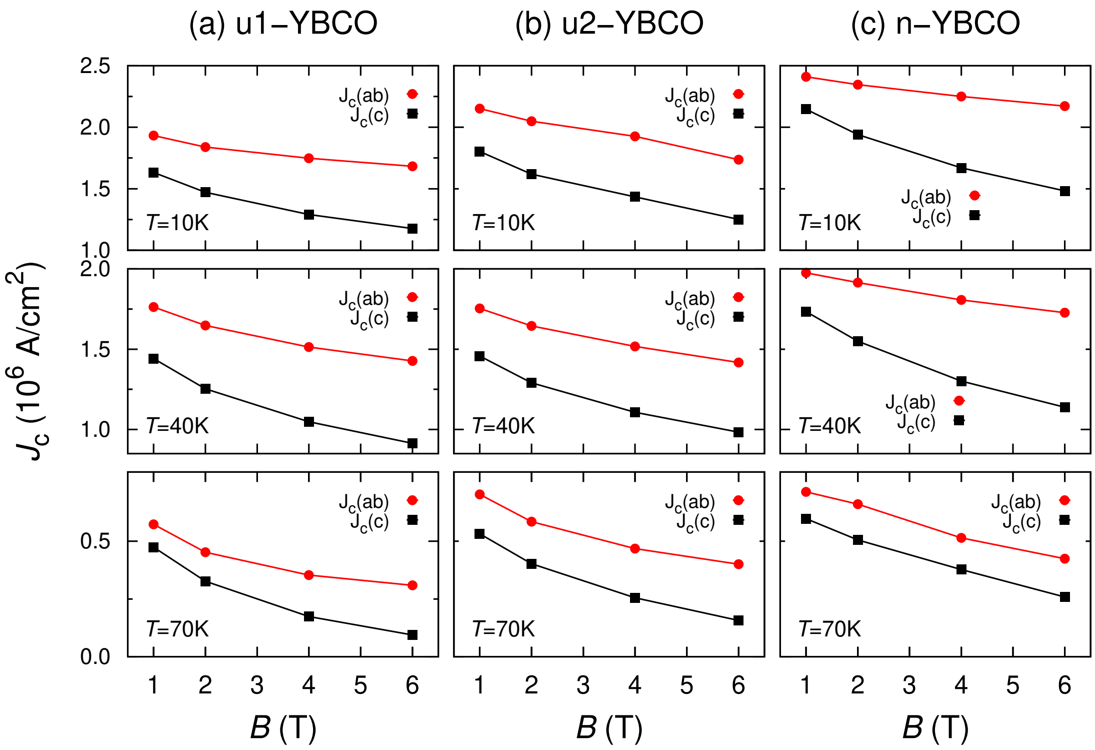

Figure S5. Comparison of absolute J_c values along the *ab*-plane and the *c*-axis in all three (a) μ1-, (b) μ2- and (c) n-YBCO films. The J_c values are given in wide magnetic field range between 1 T and 6 T and at temperatures of 10 K, 40 K and 70 K.

Table S2. The parameters used in the calculations for small and large particles, to simulate the growth in μ-YBCO and n-YBCO cases.

Parameters	Small	Large
Number of particles	150	150
Radius (nm)	0.3	50
Mass (u)	12	602
Grid size (nm x nm)	20 x 20	3334 x 3334
Time step (ns)	3	3
Drag coefficient	1e4	1e4
Leapfrog iterations	1000	1000
Statistical repeats	10	10

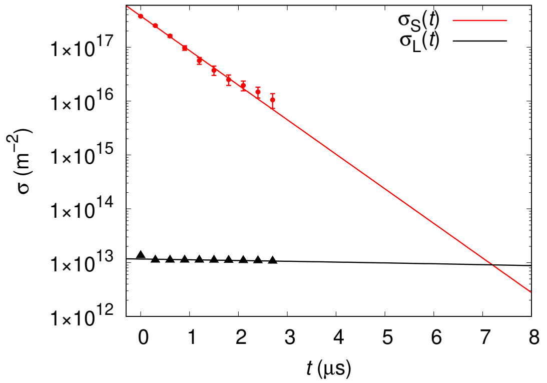

Figure S6. The simulated growth island densities with standard errors as a function of time in relatively long time scale for small (σ_S) and large (σ_L) particles as in the case of μ-YBCO and n-YBCO, respectively. The solid lines are fits to the exponential (small particles) and linear (large particles) functions.

i can be presented as

$$\text{i)}\ \ \mathbf{r}_{i+1} = \mathbf{r}_i + \mathbf{v}_i\Delta t + \frac{1}{2}(\mathbf{a}_i - \gamma\mathbf{v}_i + q\mathbf{G})\Delta t^2$$

$$\text{ii)}\ \ \mathbf{v}_{i+1/2} = \left(1-\gamma\frac{\Delta t}{2}\right)\mathbf{v}_i + (\mathbf{a}_i + q\mathbf{G})\frac{\Delta t}{2}$$

$$\text{iii)}\ \ \mathbf{a}_{i+1} = \frac{\mathbf{F}_{i+1}}{m}$$

$$\text{iv)}\ \ \mathbf{v}_{i+1} = \frac{1}{1+\gamma\frac{\Delta t}{2}}\left[\mathbf{v}_{i+1/2} + (\mathbf{a}_{i+1} + q\mathbf{G})\frac{\Delta t}{2}\right],$$

where $q = \sqrt{2\gamma kT/m}$ and γ is the drag coefficient, k is the Boltzmann constant, T is the temperature, m is the particle mass and $\mathbf{G}$ is a Gaussian random vector, whose components obey the normal distribution. Since no external force was present in the simulation, besides the drag force that is implemented inside the algorithm itself, step iii) could be skipped and steps ii) and iv) were merged together.

After each round, the positions of the particles were checked. If two particles overlapped with each other, they combined into a single particle with total area and mass equal to the sum of the collided particles. The most relevant simulation parameters are presented in Table S2. Due to computational reasons, the number of particles was set to 150 for both small and large particles. The square grid size was then chosen so that the initial surface area coverage was around 0.1.

The simulated growth island density σ as a function of time in relatively long time scale is presented in Fig. S6 with standard errors and fitted functions $\sigma_S(t) = a\exp(-bt)$ and $\sigma_L(t) = kt + c$ for small and large particles, respectively. Although the growth island density is orders of magnitude higher in the time range of the simulation, the σ_L exceeds σ_S already around t = 7 μs, according to the functions that were fitted to the data. The results are qualitatively explained by the reduced thermal motion of the larger particles due to their superior mass when compared with the smaller ones. This makes the large particles much more stable thus preventing their combination, leading finally to the greater growth island density as in n-YBCO.

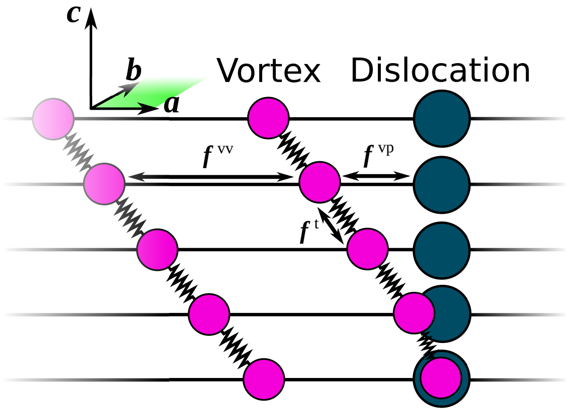

Figure S7. The schematics of the simulation model with the most relevant interaction forces: the repulsive vortex-vortex interaction f^{vv}, vortex line tension f^{t} and attractive pinning force f^{vp} between the vortex and the dislocation. The orientation of the YBCO lattice is presented on the top.

Table S3. Forces in the simulation. Parameters in the equations are vortex characteristic energy $\epsilon_0 = \Phi_0^2/(2\pi\mu_0\lambda^2) \approx 2.76\cdot 10^{11}$ J·m^{-1}, where Φ_0 is the magnetic flux quantum, the penetration depth and the coherence length along the YBCO *ab*-plane are $\lambda_{\mathrm{ab}} = 140$ nm and $\xi_{\mathrm{ab}} = 1.5$ nm, respectively, the magnetic permeability of vacuum is μ_0, the applied magnetic field is B, the normal state resistivity of YBCO is $\rho_{\mathrm{n}} \approx 5.3\cdot 10^{-7}$ Ωm, the upper critical field of YBCO in the *c*-direction at 77 K is $B_{\mathrm{c2}} \approx 27$ T, the applied current density is $\mathbf{J}$ and the direction of the vortex is $\mathbf{e}_{\mathrm{V}}$, the angular dependent Blatter scaling parameter $\varepsilon = (\sin^2(\theta)/\gamma^2 + \cos^2(\theta))$, where $\gamma \approx 5.0$ is the anisotropy parameter of YBCO and θ is the angle measured from YBCO *c*-axis, d is the distance between the adjacent vortex particles in *c*-direction and r is their distance in the *ab*-plane. The only force that acts between the adjacent layers is the vortex line tension.

Forces	Equation
Vortex line tension	$f^{\mathrm{t}} = -\dfrac{\epsilon_0 r(\gamma^2 - 1 + \ln\kappa)}{d\gamma^2\sqrt{d^2+r^2}}$
Magnetic force	$f^{\mathrm{m}} = \Phi_0\mu_0 B\sin(\theta)\dfrac{d\theta}{dr}$
Drag force	$\mathbf{f}^d = -\dfrac{\Phi_0 B_{\mathrm{n2}}}{\rho_{\mathrm{n}}}\mathbf{v}$
Lorentz force	$\mathbf{f}^{\mathrm{L}} = \mathbf{J}\times\Phi_0\mathbf{e}_{\mathrm{V}}$
Vortex-vortex interaction	$f^{\mathrm{vv}} = \dfrac{\epsilon_0}{\lambda_{\mathrm{ab}}}\mathrm{K}_1\left(\dfrac{r}{\lambda_{\mathrm{ab}}}\right)$
Pinning force	$f^{\mathrm{vp}} = \epsilon_0\dfrac{rr_0^2}{\left(r^2 + 2\varepsilon\xi_{\mathrm{ab}^2}\right)^2}$

5 Simulation model of the angular dependent J_{c}

A detailed description of the molecular dynamics simulation model and its validity has published earlier in[S7] and therefore here we summarize only the most important features of the model being relevant to understand this article. The simulation model is based on the layer structure schematically illustrated in Fig. S7, where vortices are modeled as the chains of particles connected to each other via spring-like line tension force f^{t} that acts between the two vortex particles of the same vortex located in adjacent layers. The vortex is also affected by the magnetic force f^{m} that strives to align the individual vortex particles along its angle θ. The vortex particles are also affected by the Lorentz force that strives to keep the vortices moving and drag force that slows the movement of the vortices. The attractive pinning force acts between a vortex particle and a dislocation particle that are located in the same layers while the vortex-vortex interaction repels the vortex particles of the same layer away from each another. The total force acting on vortex particle n in the layer i can thus be calculated from

$$\mathbf{F}^{\mathrm{tot}}_{i,n} = \sum_{j=i\pm1}\mathbf{f}^{\mathrm{t}}_{(i,n),(j,n)} + \sum_{j=i\pm1}\mathbf{f}^{\mathrm{m}}_{(i,n),(j,n)} + \sum_{m\neq n}\mathbf{f}^{\mathrm{vv}}_{(i,n),(i,m)} + \sum_{k}\mathbf{f}^{\mathrm{vp}}_{(i,n),(i,k)} + \mathbf{f}^{\mathrm{d}}_{(i,n)} + \mathbf{L}^{\mathrm{d}}_{(i,n)}. \quad (1)$$

The value of the critical current at certain angle was obtained iteratively by using the bisection method where the absolute value of the current was adjusted until a certain stability of the vortex system was achieved. To get good statistics, for a certain simulation five different pinning site configurations were randomly generated for which $J_{\mathrm{c}}(\theta)$s were calculated separately. The

nanorod densities were chosen to be 0.004 nm^{-2} and 0.012 nm^{-2} for μ-YBCO and n-YBCO, respectively. The final $J_c(\theta)$ was then calculated as an average value, with standard errors, of the previously mentioned simulations. Due to the layer structure of the simulation model, $J_c(\theta)$ curves can only be simulated up to $\pm 60^\circ$, since above this angle, the layer structure itself induces fictitious forces striving to align the vortex along the YBCO *c*-axis. In addition, at high angles, the repulsion forces between the vortex particles located in different layers should be taken into account which is not the case in this simulation model.

References

S1. Chrisey, D. B. & Hubler, G. K. *Pulsed Laser Deposition of Thin Films* (John Wiley Sons Inc., 1994).

S2. Peurla, M. *et al.* Optimization of the $BaZrO_3$ concentration in YBCO films prepared by pulsed laser deposition. *Supercond. Sci. Technol.* **19**, 767–771 (2006).

S3. Li, X. L., Gao, J., Wong, H. Y. & Mai, Z. H. Effect of Eu_2CuO_4/yttrium-stabilized ZrO_2 buffer layers on YBCO thin films grown on Si substrates. *Thin Solid Films* **489**, 200–204 (2005).

S4. Gauzzi, A. & Pavuna, D. Quantitative analysis of growth-induced reduction of long range lattice order in ion-beam sputtered $YBa_2Cu_3O_{6.9}$ films. *Appl. Phys. Lett.* **66**, 1836–1838 (1995).

S5. Paturi, P., Peurla, M., Nilsson, K. & Raittila, J. Crystalline orientation and twin formation in YBCO thin films laser ablated from a nanocrystalline target. *Supercond. Sci. Technol.* **17**, 564–570 (2004).

S6. Peurla, M., Huhtinen, H., Tse, Y. Y., Raittila, J. & Paturi, P. Structural properties of YBCO thin films deposited from different kinds of targets. *IEEE T. Appl. Supercond.* **17**, 3608–3611 (2007).

S7. Paturi, P., Malmivirta, M., Hynninen, T. & Huhtinen, H. Angle dependent molecular dynamics simulation of flux pinning in YBCO superconductors with artificial pinning sites. *J. Phys. Cond. Mat.* **30**, 315902:1–7 (2018).
